# Attitudes of intensivists in the UK to withdrawal of futile therapy

**DOI:** 10.1186/cc12469

**Published:** 2013-03-19

**Authors:** M Mariyaselvam, M Irvine, J Carter, M Blunt, P Young

**Affiliations:** 1 Queen Elizabeth Hospital, Kings Lynn, UK

## Introduction

We aimed to determine the current practice and attitudes of consultants in intensive care medicine when withdrawing futile life-sustaining therapy. Published guidelines suggest variation in withdrawal of futile life-sustaining therapy and are therefore not prescriptive [1]. Although there is an awareness of differing practices, the extent of these variations is not established.

## Methods

We surveyed a convenience sample of delegates at the Intensive Care Society (UK) State of the Art Meeting (2012) on attitudes and practice regarding withdrawal practice. Anonymised data were collected using surveymonkey.com.

## Results

Of 457 consultant attendees from the UK, 149 completed the survey (33%). For 58% of consultants there was no formal institutional protocol for withdrawal of futile therapy. When deciding to withdraw therapy, 57% of consultants routinely seek and document a second opinion. Regarding donation after cardiac death (DCD), 93% of consultants were happy to delay withdrawal to facilitate successful donation, 85% have already done so in their practice and 14% routinely withdraw therapy in theatres rather than on the ICU. Even if it would impact on the care of other patients, 48% would delay withdrawal of therapy to facilitate DCD. For patients accepted for DCD, 36% think that some intensivists withdraw more aggressively (in essence, hasten death) in the hope of improving the likelihood of a successful organ donation and 29% have felt pressurised to withdraw therapy more quickly than their usual practice. Furthermore, 45% experienced pressure to refer a patient for DCD when it they felt it was not appropriate.

## Conclusion

This survey confirms variation in the practice and attitudes to withdrawal of futile therapy amongst UK consultant intensivists. Formal protocols were frequently unavailable to guide withdrawal and second opinions were often not sought. Nearly one-half of the intensivists delay withdrawal to facilitate donation, even if this may impact on the care of other patients. Many intensivists have felt pressure to refer for donation when they feel this is inappropriate and there is a perception that some intensivists may withdraw care more aggressively in those who are accepted for DCD to improve the likelihood of a successful donation. This survey may help inform debate in this ethically challenging area.
